# Meta-analysis of insulin aspart versus regular human insulin used in a basal–bolus regimen for the treatment of diabetes mellitus

**DOI:** 10.1111/1753-0407.12060

**Published:** 2013-05-07

**Authors:** Simon Heller, Bruce Bode, Plamen Kozlovski, Anne Louise Svendsen

**Affiliations:** 1 School of Medicine and Biochemical SciencesSheffield, UK; 2 Emory University School of MedicineAtlanta, Georgia, USA; 3 Novo Nordisk A/SBagsvaerd, Denmark

**Keywords:** human blood A1c protein, hypoglycemia, insulin aspart, regular human insulin, meta-analysis.

## Abstract

**Background:**

The objective of the current study was to compare the efficacy of two different insulin formulations, insulin aspart (IAsp) and regular human insulin (RHI), for prandial insulin coverage with neutral protamine Hagedorn (NPH) insulin as basal insulin using a meta-analysis approach. The primary endpoint was change in A1c over time. Secondary endpoints included incidence of hypoglycemia and postprandial glycemic control.

**Methods:**

Clinical trials (Type 1 and Type 2 diabetes) complying with Good Clinical Practice, and with individual patient data, were included in the meta-analysis. Trials were randomized, consisting of (at least) two treatment arms and had a minimum duration of 12 weeks. Estimates were calculated using fixed-effects and random-effects models. Heterogeneity was assessed for each analysis. The effect of baseline parameters on A1c was analyzed in extended simultaneous models.

**Results:**

The mean difference in A1c was 0.1% (95% confidence interval [CI] [−0.15; −0.04], *P* < 0.001) in favor of IAsp. Higher accumulated dose of IAsp, higher age and increased rates of hypoglycemia were associated with improved A1c outcome. Fasting plasma glucose was not significantly different between regimens. Postprandial glucose was significantly lower after treatment with IAsp compared with RHI, but the analysis did present a significant level of heterogeneity (*P* < 0.001). The overall rate of hypoglycemia was the same with both regimens, but nocturnal hypoglycemia was significantly lower with IAsp.

**Conclusions:**

A basal–bolus regimen with IAsp as bolus insulin provided minimal, but statistically significant, improvement in overall glycemic control with a lower rate of nocturnal hypoglycemic episodes, compared with a corresponding regimen with bolus RHI.

**Significant findings of the study:** After 12 weeks of treatment, IAsp + NPH significantly reduced mean HbA_1c_, prandial blood glucose increment and the risk for nocturnal hypoglycemia, compared with RHI + NPH.

**What this study adds:** Our findings reveal that IAsp + NPH demonstrates minimal glycemic control improvement versus RHI + NPH; its main benefit is in reducing the nocturnal hypoglycemia risk by about 25%, a finding rarely reported in systematic reviews and meta-analyses.

## Introduction

In people with diabetes, insulin aspart (IAsp) (NovoRapid, Novo Nordisk, Denmark) is more rapidly absorbed than regular human insulin (RHI), with higher maximal concentration and shorter time to peak concentration.^1^–^3^ However, total absorption, the area under the concentration–time curve, is similar for IAsp and RHI. Glucose-clamp studies have confirmed that the reported pharmacokinetic profile for IAsp also reflects differences in pharmacodynamic activity, showing a significantly greater and faster glucose-lowering effect compared with RHI.^1^–^3^ IAsp has been extensively studied in clinical trials as the bolus insulin in basal–bolus regimens and has consistently shown improved postprandial glycemic control compared with RHI in both Type 1 and Type 2 diabetes.^4^–^7^ In some of these studies, a small but statistically significant difference in A1c was found in favor of IAsp,^3^ whereas in other studies, A1c levels were comparable.^4^–^7^

The aim of this meta-analysis was to compare IAsp with RHI, both used as bolus insulin in basal–bolus therapy, with respect to glycemic control. Neutral protamine Hagedorn (NPH) insulin was used as the basal component in both regimens. The primary endpoint of this analysis was long-term glycemic control, as measured by change in A1c over time. Secondary endpoints included incidence of hypoglycemia and postprandial glycemic control.

## Methods

### Trial selection

This retrospective meta-analysis was performed according to the guidelines of Whitehead^8^ and The Cochrane Policy Manual.^9^ Trials were considered for inclusion if they complied with Good Clinical Practice guidelines (in order to limit heterogeneity and ensure quality of data), demonstrated methodological quality, included IAsp in one of the treatment arms and had individual patient data available. Seventy-eight trials fulfilled the above conditions, of which, 10 trials satisfied the inclusion and exclusion criteria listed below and were therefore included in the final analysis.

Inclusion criteria:

1. Randomized and controlled trials.
2. Trials had to consist of (at least) two arms with IAsp and RHI, respectively, as bolus insulin, and with NPH as the intermediate-acting basal insulin in both arms.
3. Trials had to have a treatment period of at least 12 weeks. This period was chosen to collect meaningful data on efficacy for the primary endpoint.

Exclusion criteria:

1. Trials with pregnant women or children (<18 years).

Two trials had a crossover design; from these studies, only data from the first treatment period were included in the meta-analysis. Five of the included studies have been published.^5^,^10^,^11^–^13^

### Statistical methods

Estimates of the treatment effects were calculated for every trial included in the meta-analysis. These estimates were combined into overall estimates using a fixed-effects model (in which the estimates of the individual trials are considered similar enough to assume that they are all estimates of a common treatment effect) and a random-effects model (in which individual trial estimates are treated as independent samples from a normal distribution) to account for heterogeneity. A fixed-effects model may be considered an implausible oversimplification,^14^ whereas a random-effects model is considered to be more complex, realistic and conservative.^15^ Heterogeneity was measured using the weighted sum of squares test, thus it was assumed that the treatment effects were distributed homogenously; that is, the difference between each treatment effect estimate and the overall treatment effect estimate were normally distributed. Values were estimated using the intention-to-treat population of each trial.

### Efficacy analyses

The primary endpoint was the change in A1c over time. In the A1c analysis, the possible influence of predictors such as treatment, demographic characteristics, insulin dose, age and incidence of hypoglycemic episodes were evaluated. Secondary endpoints included change in fasting blood glucose (FBG) and change in postprandial blood glucose (PPG).

### A1c analysis – per-trial model

A1c was measured repeatedly over time and analyzed in a linear mixed model. The time trends were estimated separately for the two treatment arms using all available measurements. Since different time trends would be expected before and after week 12,^16^,^17^ the time trend was modeled as a linear spline with a knot at week 12. The treatment effect arising from the difference between these two splines depended on the reporting time points; we chose to report the result of the analysis at both week 12 and week 24. In addition to the time trend, the model included baseline A1c (corresponding to the intercept) and random slopes (before and after week 12) for each subject. Given the limited amount of data, a simple covariance structure of the two random slopes was used, assuming that they had different variance and were uncorrelated.

### A1c analysis – simultaneous models

A simple simultaneous model was constructed by extending the per-trial model and pooling all trial data. The model included the interaction of trial and time, and used an unstructured covariance of the two random slopes, allowing correlation between them. An extended simultaneous model was also constructed by incorporating several covariates into the simple simultaneous model; these included accumulated bolus insulin dose (daily dose × days of treatment), age, body mass index, diabetes duration and frequency of hypoglycemic episodes. Trials 066, 1266 and 1634 were excluded from the simultaneous model as they did not have repeated measurements of A1c and therefore did not fit the meta-analysis framework.

### Fasting blood glucose

As for A1c, analyses for FBG were performed using a spline with a knot at week 12 and results were reported at 12 and 24 weeks. Given the limited amount of data, this analysis was supplemented with an analysis using only one linear trend, reported at week 16.

### Postprandial blood glucose

Postprandial blood glucose was calculated as the mean of the post-meal measurements (90 min after each meal) after breakfast, lunch and dinner. PPG increment was the corresponding mean difference between pre- and post-meal blood glucose.

### Insulin dose

No formal statistical analysis was undertaken specifically for insulin dose or change in insulin dose during treatment. However, in the extended A1c model, the relationship between A1c and accumulated bolus insulin dose was reported.

### Safety analyses

Safety analyses were limited to hypoglycemic episodes. The number of hypoglycemic episodes was modeled using a negative binomial regression model. Effect estimates were calculated as rate ratios: the rate of hypoglycemic episodes for patients treated with IAsp relative to the rate of episodes for patients treated with RHI. The model was run separately for each trial, and the overall fixed-effects and random-effects estimates were calculated. Episodes were only included in the analyses if they were considered treatment-emergent that is, if they occurred on or between the dates of the first and the last doses. Hypoglycemic episodes were classified into three groups: major, minor and symptoms-only (episodes not falling within any of the three categories were “unclassified”). The term “all” was used to denote all hypoglycemic episodes. The term “major” was used for any episode in which the subject was unable to treat him- or herself. Episodes handled by the subjects themselves were classified as “minor” when confirmed by blood glucose measurements <2.8 mmol/L, and as “symptoms-only” when blood glucose was ≥2.8 mmol/L or the measurement was missing. In some trials, glucose measurements were reported as blood glucose, while, in others, they were reported as plasma glucose. All values presented here were converted to blood glucose. Nocturnal hypoglycemic episodes were defined as hypoglycemic episodes occurring in the time interval 00.00 h to 06.00 h, inclusive. Nocturnal episodes were only analyzed within the “all” class of episodes.

Hypoglycemia data from one trial (054) were not included in the safety analyses as it did not provide relevant information with regard to the classification of episodes, measures of blood glucose and whether the subject was able to treat him- or herself.

## Results

The analysis included 10 trials, eight parallel-design and two crossover trials, conducted in subjects with Type 1 diabetes (six trials), Type 2 diabetes (three trials) or both types (one trial). Most of the trials were multinational (7/10). Trial characteristics are presented in Table 1.

**Table 1:** Baseline characteristics of the trial populations – ITT

Trial (reference)	Duration (weeks)	Location	*n*		Mean age (SD) *years*		Sex *(% F/M)*		Mean duration of diabetes (SD) *years*		Mean A1c pre-trial (SD) (%)		Mean total dose (SD) *(U/kg)*	
			IAsp	RHI	IAsp	RHI	IAsp	RHI	IAsp	RHI	IAsp	RHI	IAsp	RHI
*Type 1 diabetes*														
035 (Home 2000)^5^	24	Multi-national	707	358	37.6 (11.2)	37.9 (11.9)	45.4/54.6	44.1/55.9	14.8 (10.1)	15.1 (10.1)	8.0 (1.2)	8.0 (1.2)	0.71 (0.20)	0.69 (0.22)
036 (Raskin 2000)^10^	24	Multi-national	596	286	38.9 (10.5)	39.9 (12.2)	48.7/51.3	46.9/53.1	15.8 (9.7)	15.9 (9.3)	7.9 (1.1)	8.0 (1.3)	0.71 (0.26)	0.69 (0.24)
054[Table-fn tf1-1]	24	Japan	130	56	36.6 (14.8)	36.4 (13.4)	56.9/43.1	66.1/33.9	11.4 (7.1)	11.8 (7.0)	7.5 (1.1)	7.5 (1.1)	0.77 (0.24)	0.85 (0.29)
064 (DeVries 2003)^11^	64	Multi-national	186	181	38.5 (12.8)	38.8 (12.8)	37.6/62.4	38.7/61.3	15.5 (9.7)	17.8 (11.1)	8.4 (0.8)	8.4 (0.8)	0.80 (0.24)	0.75 (0.21)
065 (Tamás 2001)^12^	64	Multi-national	211	212	35.6 (11.4)	36.0 (11.7)	41.7/58.3	45.3/54.7	14.0 (9.1)	14.3 (9.0)	8.4 (0.9)	8.3 (0.8)	0.78 (0.24)	0.76 (0.20)
066†	16‡	Multi-national	79	76	38.0 (12.4)	39.4 (10.2)	38.0/62.0	39.5/60.5	15.4 (10.7)	14.5 (8.8)	7.8 (0.8)	7.8 (0.8)	0.76 (0.23)	0.74 (0.25)
*Type 2 diabetes*														
037†	24	Multi-national	91	91	56.6 (9.8)	58.3 (10.0)	37.4/62.6	39.6/60.4	12.8 (7.7)	13.0 (8.0)	8.1 (1.2)	7.9 (1.1)	0.64 (0.26)	0.66 (0.30)
1198†	16	Multi-national	87	89	63.5 (9.2)	61.8 (9.3)	49.4/50.6	50.6/49.4	14.8 (7.6)	14.3 (7.4)	9.7 (1.3)	10.1 (1.3)	0.59 (0.23)	0.60 (0.26)
1266†	16‡	Italy	41	30	61.3 (7.3)	60.8 (7.1)	68.3/31.7	56.7/43.3	14.8 (6.3)	17.2 (7.0)	8.7 (0.7)	8.9 (0.8)	0.60 (0.25)	0.66 (0.28)
*Type 1 and 2 diabetes*														
1634§ (Gao 2009)^13^	12	China§	110	110	51.8 (8.8)	52.9 (7.8)	53.6/46.4	53.6/46.4	6.9 (6.1)	9.3 (5.2)	9.3 (1.4)	9.2 (1.2)	0.64 (0.26)	0.66 (0.24)
All trials¶			2238	1489	40.7 (13.3)	42.4 (14.1)	46.3/53.7	45.8/54.2	14.4 (9.5)	14.7 (9.4)	8.1 (1.2)	8.3 (1.2)	0.71 (0.23)	0.71 (0.24)

† Unpublished data.

‡ Crossover trial with two periods of 16 weeks; only data from period 1 used in the meta-analysis.

§ 15 Type 1 and 205 Type 2 diabetes.

¶ 3093 Type 1 and 634 Type 2 diabetes. In all trials, 6.8% and 8.1% of patients did not complete treatment with IAsp or RHI, respectively.

IAsp, insulin aspart; RHI, regular human insulin; SD, standard deviation.

### Efficacy analyses

**Figure 1:**
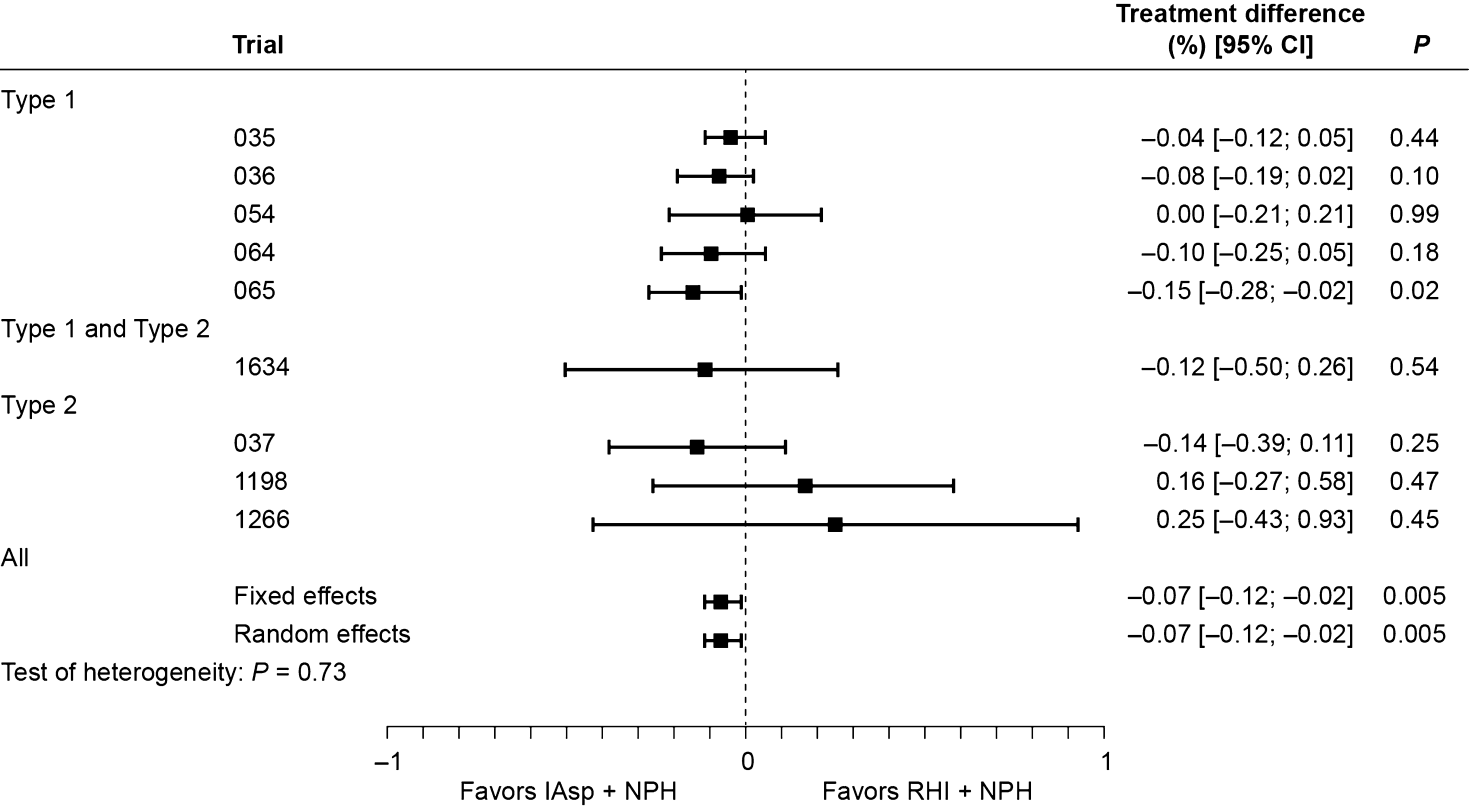
Change in A1c (%): per-trial and overall analysis of treatment difference at week 12.[Correction added on 7 June 2013, after first online publication: The last two *P*-values have been corrected from 0.01 to 0.005.]

The 12-week analysis results for A1c (per-trial model, excluding trial 066) are shown in Fig. 1. The overall estimate for A1c was significantly lower with IAsp compared with RHI, although the difference was small (−0.07%, 95% confidence interval [CI] [–0.12%; –0.02%]; *P* = 0.005); heterogeneity was low (*P* = 0.730), and the estimates achieved with the fixed-effects and the random-effects models were identical. The overall estimate for A1c after 24 weeks was similar (–0.11%, 95% CI, [–0.17%; –0.06%]; *P* < 0.001) and also presented low heterogeneity (*P* = 0.952). In trial 066, HbA_1c_ was measured only at baseline and after 16 weeks; therefore, it was not included in the efficacy analysis.

### Simultaneous A1c analysis model

The simple simultaneous model (excluding trials 066, 1634 and 1266, as they did not have repeated measurements of A1c) yielded a similar overall estimate (–0.1%, 95% CI [–0.15; –0.04]; *P* < 0.001) as the per-trial model. The decrease in A1c occurred primarily in the first 12-week period (data not shown). Adding baseline A1c values to the model did not affect the overall treatment difference estimates, but confirmed that glycemic improvement occurred shortly after starting treatment (Table 2). An inter-subject difference of 1% at baseline would have been reduced to 0.76% after 12 weeks of treatment, and after an additional 12 weeks to 0.74%. Thus, beyond the first 12 weeks of treatment, the influence of baseline A1c level on the decrease in A1c over time was very small.

**Table 2:** Change in A1c (%): simultaneous models*

Factors	Period	Estimated change in A1c (95% CI)	*P*-value
*Simple model*			
Treatment	12 weeks	−0.10 [−0.15; −0.04]	<0.001
Treatment	24 weeks	−0.10 [−0.15; −0.04]	<0.001
*Simple model plus A1c at baseline*			
Treatment	12 weeks	−0.10 [−0.15; −0.04]	<0.001
Treatment	24 weeks	−0.10 [−0.15; −0.05]	<0.001
Baseline A1c (per 1% difference)	12 weeks	0.76 [0.73; 0.78]	<0.001
Baseline A1c (per 1% difference)	24 weeks	0.74 [0.71; 0.76]	<0.001
*Extended model*^†^			
Treatment	12 weeks	−0.04 [−0.11; 0.04]	0.333
Treatment	24 weeks	0.02 [−0.08; 0.13]	0.696
Baseline A1c (per 1% difference)	12 weeks	0.76 [0.73; 0.78]	<0.001
Baseline A1c (per 1% difference)	24 weeks	0.74 [0.72; 0.76]	<0.001
Accumulated IAsp (per 50 U/Kg)		−0.09 [−0.14; −0.05]	<0.001
Accumulated difference (IAsp–RHI)		−0.09 [−0.15; −0.02]	0.007
Age (per 10 years)	12 weeks	−0.03 [−0.06; −0.01]	0.006
Age (per 10 years)	24 weeks	−0.05 [−0.07; −0.03]	<0.001
Hypoglycemia (per 10 episodes)		−0.02 [−0.03; −0.01]	<0.001

* Excluding trials 066, 1266 and 1634.

† Accounting for effect of HbA_1c_ at baseline, accumulated bolus dose, age and hypoglycemic episodes.

IAsp, insulin aspart; RHI, regular human insulin.

The estimates derived from the extended simultaneous model are shown in the lower part of Table 2. The accumulated dose of IAsp and the accumulated bolus dose difference (IAsp−RHI) were both significant predictors for change in A1c, suggesting that increasing the IAsp dose (in absolute terms or relative to RHI) would predict an increased difference between regimens and a decrease in A1c. The accumulated dose of NPH was not a significant predictor for change in A1c (data not shown). When accumulated dose is included in the model, the effect of treatment is no longer significant, as “accumulated dose” includes the interaction with time. As may be expected, a rise in the number of hypoglycemic episodes corresponded to a further lowering of A1c. Age alone was found to be a significant A1c predictor; that is, the older the person was at baseline, the more A1c was likely to improve during treatment. The addition of age and accumulated hypoglycemic episodes (number of episodes from baseline) to the model had very little, if any, impact on the effect of baseline A1c. Similar results were obtained when all 10 trials were included in the extended model (data not shown).

### Fasting blood glucose

Fasting blood glucose was inconsistently reported in the selected trials; only six trials had relevant data before and after week 12. No significant treatment difference was detected in the analysis, either at week 12 or at week 24, but heterogeneity was high (*P* = 0.013). Similar results were obtained when the analysis was repeated for the 16-week treatment period with inclusion of data from all 10 trials.

### Postprandial blood glucose

**Figure 2:**
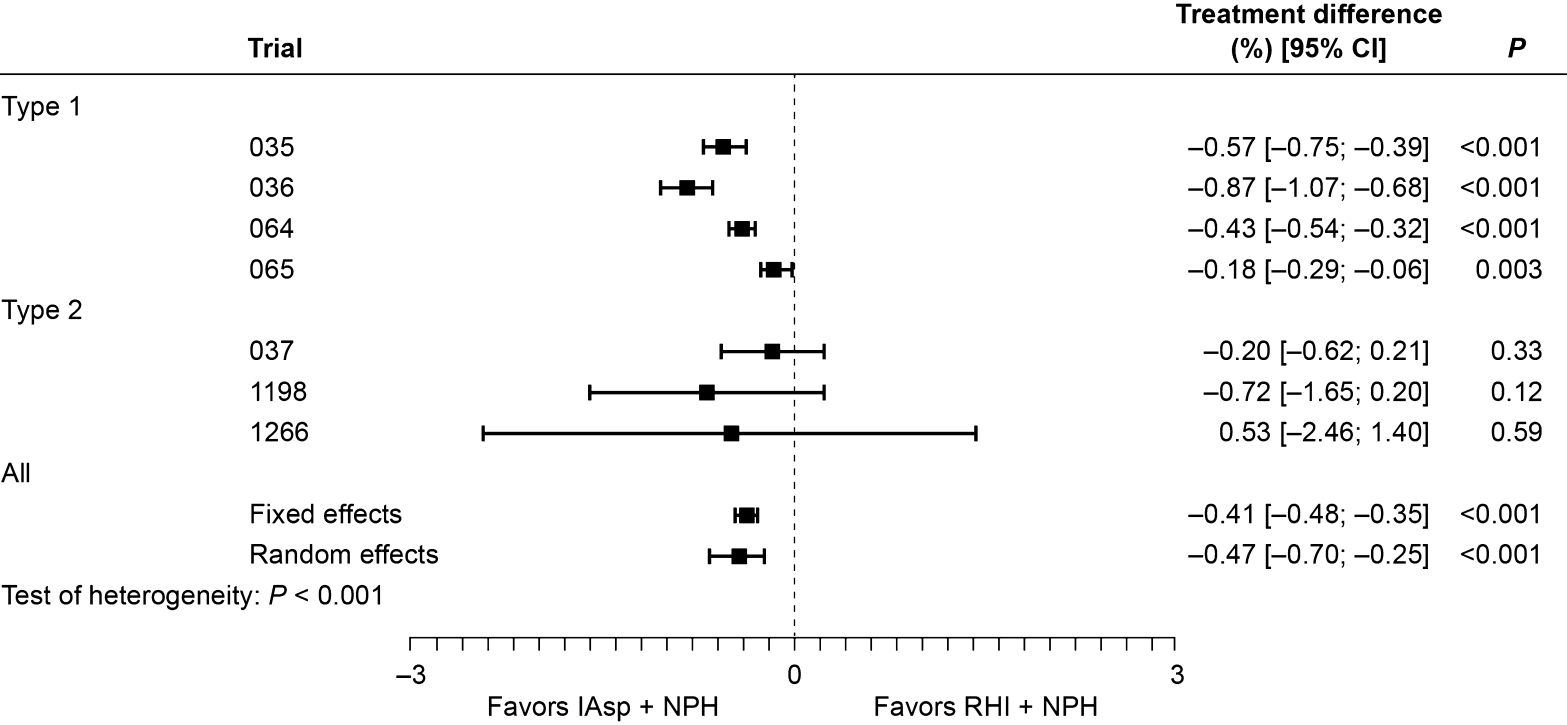
Change in average blood glucose level (mmol/L) after breakfast, lunch and dinner: per-trial and overall analysis of treatment differences at week 16.

Only the 16-week collection period provided relevant data for the meta-analysis of PPG. A significant difference was reported between regimens in favor of IAsp, with overall estimates of –0.41 mmol/L (95% CI [–0.48; –0.35]; *P* < 0.001) and –0.47 mmol/L (95% CI [–0.70; –0.25]; *P* < 0.001) using the fixed-effects and the random-effects analysis models, respectively (Fig. 2). Given the significant level of heterogeneity within this dataset (*P* < 0.001), the random-effects model estimate was considered more appropriate and, in this case, still demonstrated a significant difference between regimens. The analysis of the average change in PPG increment gave similar results. The difference between regimens was significant in favor of IAsp and, although the dataset was heterogeneous (*P* ≤ 0.001), both models showed significant differences with a fixed-effects estimate of –0.45 mmol/L (95% CI [–0.51; –0.38]; *P* < 0.001) and a random-effects estimate of –0.61 mmol/L (95% CI [–0.88; –0.34]; *P* < 0.001).

### Safety analyses

**Figure 3:**
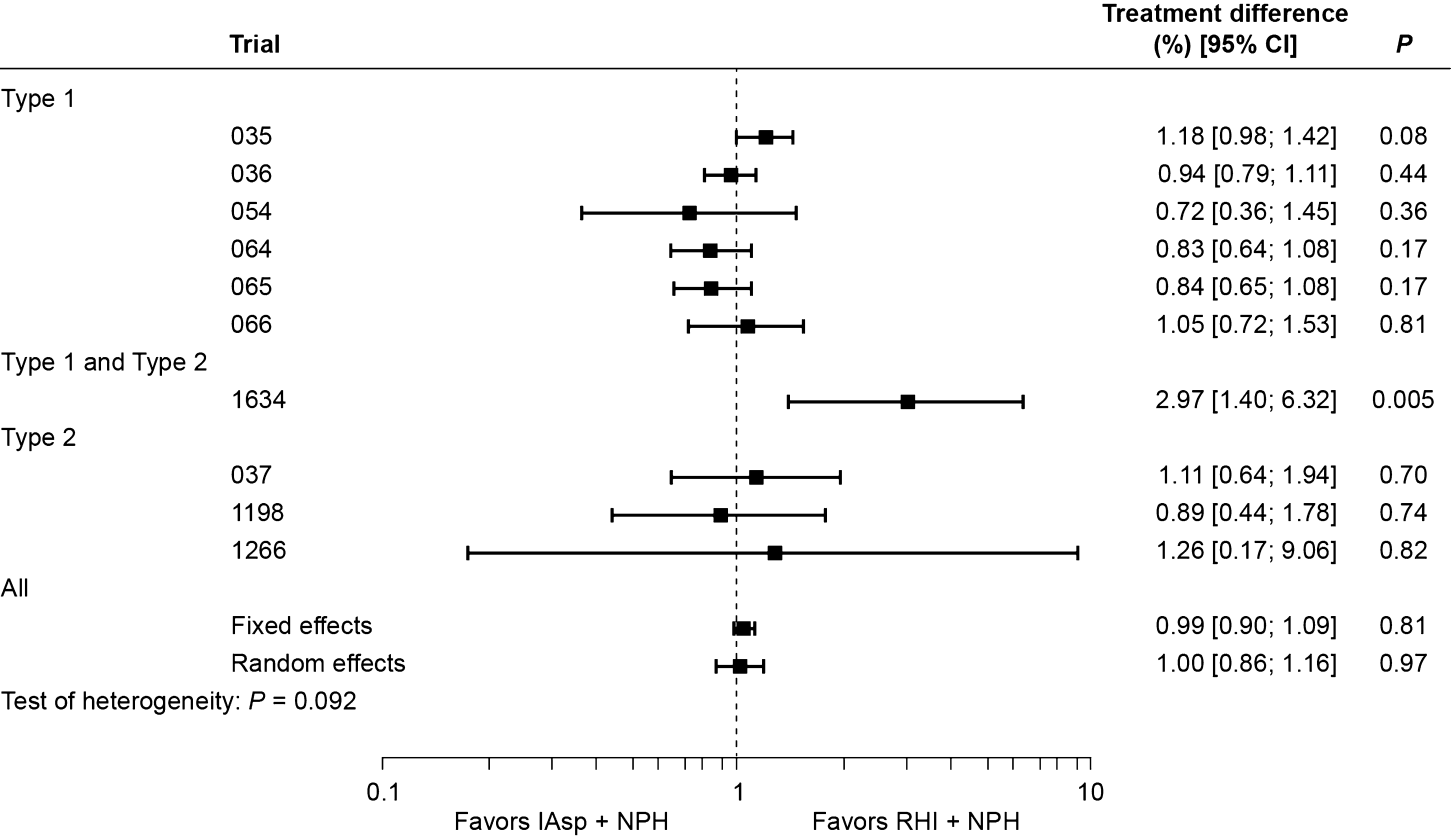
Per-trial and overall analysis of all hypoglycemic episodes (rate ratios with 95% confidence intervals).[Correction added on 7 June 2013, after first online publication: The *P*-value for ‘Type 1 and Type 2’ has been corrected from 0.01 to 0.005.]

**Figure 4:**
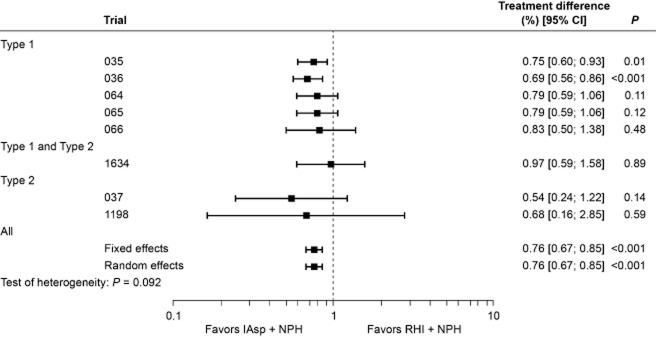
Per-trial and overall analysis of nocturnal hypoglycemic episodes (rate ratios with 95% confidence intervals).

The rate of “all” hypoglycemic episodes varied widely between trials. In general, the rate of hypoglycemia was higher in subjects with Type 1 diabetes compared with those with Type 2 diabetes (Table 3). This difference was found with all types of episode (major, minor and symptoms-only; data not shown). The overall (and per-trial) analysis of “all” hypoglycemic episodes is shown in Fig. 3. Estimates from the fixed-effects (0.99; 95% CI [0.90; 1.09], *P* = 0.813) and random-effects analyses (1.00; 95% CI [0.86; 1.16], *P* = 0.970) indicated that the overall rate of episodes was comparable for the two regimens, although the data were heterogeneous (*P* = 0.055). A separate analysis made for diurnal episodes showed a similar trend, with no difference between regimens (data not shown). By contrast, heterogeneity was low (*P* = 0.924) for nocturnal episodes, and the rate of nocturnal hypoglycemia was significantly lower with IAsp compared with RHI (rate ratio for fixed- and random-effect estimate was 0.76, 95% CI [0.67; 0.85], *P* < 0.001; Fig. 4).

**Table 3:** All treatment-emergent and all nocturnal treatment-emergent hypoglycemic episodes by treatment and trial

Trial	Type of diabetes	All treatment-emergent hypoglycemic episodes								All nocturnal treatment-emergent hypoglycemic episodes							
		IAsp				RHI				IAsp				RHI			
		*n*	(%)	E	Rate	*n*	(%)	E	Rate	*n*	(%)	E	Rate	*n*	(%)	E	Rate
035	1	572	80.9	10 430	30.0	278	77.7	4 480	26.0	361	51.1	1520	4.4	205	57.3	1011	5.9
036	1	536	89.9	13 019	45.2	249	87.1	6 521	47.5	357	59.9	1545	5.4	190	66.4	1030	7.5
054	1	48	36.9	300	5.1	29	51.8	178	7.0	–	–	–	–	–	–	–	–
064	1	167	89.8	5 129	24.1	166	91.7	6 167	29.5	127	68.3	796	3.7	134	74.0	1029	4.9
065	1	193	91.5	9 037	36.9	193	91.0	10 824	44.8	154	73.0	1494	6.1	155	73.1	1870	7.7
066	1	73	92.4	1 044	43.6	67	88.2	1 005	41.6	38	48.1	132	5.5	47	61.8	163	6.7
1266	2	5	12.2	7	0.7	1	3.3	4	0.6	–	–	–	–	1	3.3	1	0.2
037	2	57	62.6	785	17.6	58	63.7	686	15.9	22	24.2	97	2.2	29	31.9	177	4.1
1198	2	30	34.5	122	4.6	32	36.0	145	5.2	6	6.9	9	0.3	6	6.7	14	0.5
1634	1 & 2	26	23.6	73	2.9	17	15.5	25	1.0	3	2.7	3	0.1	4	3.6	5	0.2
All		1707	76.3	39 946	31.1	1 090	73.2	30 035	32.9	1068	47.7	5596	4.4	771	51.8	5300	5.8

%, proportion of subjects in the population having hypoglycemic episodes; E, number of hypoglycemic episodes; IAsp, insulin aspart; *n*, number of subjects; rate: episodes per subject year of exposure in the population; RHI, regular human insulin.

## Discussion

Previously published meta-analyses comparing the efficacy and safety of rapid-acting insulin analogs with RHI have consistently shown a modest but statistically significant difference in A1c in patients with Type 1 or Type 2 diabetes, in favor of rapid-acting analogs.^18^–^20^ These meta-analyses were based on systematic reviews of published trial data. Our meta-analysis was based on data from randomized controlled trials in patients with Type 1 or Type 2 diabetes that included individual patient data; to date, only five of the included trials have been published.^5^,^10^–^13^ Our findings confirm that IAsp + NPH treatment improves overall glycemic control (A1c) and PPG compared with RHI + NPH treatment. The improvement in A1c was small and thus clinically of marginal benefit. By contrast, FBG showed no between-treatment difference. This was to be expected, as FBG is mainly controlled by the level of the basal insulin component, which was the same for both regimens.

Importantly, improved glycemic control should not be achieved at the expense of an unacceptable increase in the risk for hypoglycemia. Data published from previous meta-analyses did not reveal differences in overall hypoglycemia when comparing rapid-acting analogs and RHI;^20^ however, due to a high degree of heterogeneity, it may have been difficult to draw any firm conclusions. Our analysis confirmed this finding for overall and diurnal hypoglycemia. However, heterogeneity was low for the analysis of nocturnal hypoglycemia, and event rates were significantly lower with IAsp compared with RHI. The lower overall incidence of nocturnal hypoglycemia with IAsp, as observed in some of the individual trials and reported with other rapid-acting analogs,^21^ is to be expected, as IAsp is eliminated considerably faster than RHI from circulation. Nonetheless, a reduction in the rate of nocturnal hypoglycemia, a variable rarely investigated in systematic reviews, may have important clinical implications that should not be overlooked. Studies show that nocturnal hypoglycemia can have a detrimental effect on health, quality of life and productivity in patients with Type 1 or Type 2 diabetes.^22^,^23^ Patients who experience nocturnal hypoglycemic episodes report an increase in tiredness, sleeping difficulties and absenteeism from work. In addition, some patients reduce their normal insulin dose after an episode, thus potentially compromising their diabetes management regimen.^24^ It is also recognized that patients substantially fear the recurrence of nocturnal hypoglycemia, which may reduce their adherence to insulin therapy.^23^ Furthermore, nocturnal hypoglycemia may also contribute toward (i) the impaired awareness of future hypoglycemic events, which, in turn, may increase the risk of major hypoglycemia,^25^,^26^ and (ii) terminal conditions, such as “dead-in-bed” syndrome.^27^

Most published data, whether from individual trials or meta-analyses, are based on non-treat-to-target regimens. In these studies, the increase in insulin dose during treatment was moderate, and the absolute treatment effect on A1c was small. Our study supports these findings; however, the accumulated dose of IAsp (but not of RHI) was a significant predictor for A1c, indicating that an increase in IAsp dose, absolute or relative to RHI, would predict an increased difference between the two regimens. In a treat-to-target designed study, up-titration of IAsp to predetermined glycemic targets may result in a greater reduction of A1c; however, in turn, higher doses of IAsp may also increase the risk of hypoglycemia. In relative terms, another significant predictor of A1c was age: the older the person was at baseline, the more A1c was likely to improve during treatment, independent of treatment regimen.

Important limitations of our meta-analysis include the short duration of most of the trials and that none of them had a treat-to-target design. The meta-analysis may also have been confounded by the different study designs/protocols of the included trials; however, this is an inherent problem of meta-analyses and we have attempted to account for heterogeneity where possible. In addition, as the main focus of the study was to compare two different formulations of bolus insulin, it is possible that the titration of basal insulin was not monitored as robustly as bolus titration. It remains to be established how the two treatment regimens would compare in trials of longer duration and a more aggressive treatment design, in particular with respect to glycemic control and hypoglycemia, and the relationship between the two. Given a basal–bolus regimen and a treat-to-target titration algorithm for the basal and meal-related insulin, it might be possible to fine-tune the total insulin dose according to individual characteristics, such as stage of disease, age and level of physical activity. Therefore, it might be possible to maximize the different pharmacokinetic and pharmacodynamic properties of IAsp, through dose optimization, to improve glycemic control while minimizing the risk of hypoglycemia.

In conclusion, this meta-analysis shows that IAsp used in combination with NPH insulin provides a minimal but statistically significant improvement in glycemic control compared with RHI and NPH. Furthermore, IAsp was significantly associated with a clinically meaningful and statistically significant reduction in nocturnal hypoglycemia – a variable not often reported in systematic reviews yet linked with several aspects of diabetes management, such as treatment adherence, patients' quality of life and the overall risk of hypoglycemia.
